# In silico evaluation of a targeted metaproteomics strategy for broad screening of cellulolytic enzyme capacities in anaerobic microbiome bioreactors

**DOI:** 10.1186/s13068-022-02125-x

**Published:** 2022-03-18

**Authors:** Manuel I. Villalobos Solis, Payal Chirania, Robert L. Hettich

**Affiliations:** 1 Biosciences Division, Oak Ridge National Laboratory, Oak Ridge, TN 37831 USA; 2 UT-ORNL Graduate School of Genome Science and Technology, University of Tennessee, Knoxville, TN 37996 USA

**Keywords:** Biogas, Glycoside hydrolases, Peptides, Lignocellulose, Anaerobic digester, Targeted metaproteomics, Microbial community, Microbiome

## Abstract

**Background:**

Microbial-driven solubilization of lignocellulosic material is a natural mechanism that is exploited in anaerobic digesters (ADs) to produce biogas and other valuable bioproducts. Glycoside hydrolases (GHs) are the main enzymes that bacterial and archaeal populations use to break down complex polysaccharides in these reactors. Methodologies for rapidly screening the physical presence and types of GHs can provide information about their functional activities as well as the taxonomical diversity within AD systems but are largely unavailable. Targeted proteomic methods could potentially be used to provide snapshots of the GHs expressed by microbial consortia in ADs, giving valuable insights into the functional lignocellulolytic degradation diversity of a community. Such observations would be essential to evaluate the hydrolytic performance of a reactor or potential issues with it.

**Results:**

As a proof of concept, we performed an in silico selection and evaluation of groups of tryptic peptides from five important GH families derived from a dataset of 1401 metagenome-assembled genomes (MAGs) in anaerobic digesters. Following empirical rules of peptide-based targeted proteomics, we selected groups of shared peptides among proteins within a GH family while at the same time being unique compared to all other background proteins. In particular, we were able to identify a tractable unique set of peptides that were sufficient to monitor the range of GH families. While a few thousand peptides would be needed for comprehensive characterization of the main GH families, we found that at least 50% of the proteins in these families (such as the key families) could be tracked with only 200 peptides. The unique peptides selected for groups of GHs were found to be sufficient for distinguishing enzyme specificity or microbial taxonomy. These in silico results demonstrate the presence of specific unique GH peptides even in a highly diverse and complex microbiome and reveal the potential for development of targeted metaproteomic approaches in ADs or lignocellulolytic microbiomes. Such an approach could be valuable for estimating molecular-level enzymatic capabilities and responses of microbial communities to different substrates or conditions, which is a critical need in either building or utilizing constructed communities or defined cultures for bio-production.

**Conclusions:**

This in silico study demonstrates the peptide selection strategy for quantifying relevant groups of GH proteins in a complex anaerobic microbiome and encourages the development of targeted metaproteomic approaches in fermenters. The results revealed that targeted metaproteomics could be a feasible approach for the screening of cellulolytic enzyme capacities for a range of anaerobic microbiome fermenters and thus could assist in bioreactor evaluation and optimization.

**Supplementary Information:**

The online version contains supplementary material available at 10.1186/s13068-022-02125-x.

## Background

The solubilization of lignocellulosic waste material (i.e., woody biomass and municipal solid waste) holds great potential for the generation of biogas and other valuable bioproducts [1]. This process can be achieved by employing anaerobic digestion by microbes which break down complex organic material to generate a variety of end-products, including biogas [2–4]. Amongst the metabolic steps performed by microbes during this conversion, the hydrolysis of constituent polysaccharides is considered an essential and rate-limiting step [5–7]. The success of anaerobic digesters (ADs) to utilize complex biomass thus depends on the activity of (ligno-)cellulolytic or hydrolytic bacteria and their repertoire of glycoside hydrolases (GHs) and other Carbohydrate-Active enZYmes (CAZymes) [5, 8, 9]. Therefore, to improve and maintain hydrolysis efficiencies, it is important to understand/identify the type of lignocellulose-degrading microorganisms that thrive in diverse bioreactor environments and gain information about their metabolism.

The integrated application of multiple omics approaches has enabled a deep understanding of the metabolic potential of microbial communities and their function within ADs. Among these approaches, metaproteomic investigations in ADs have identified and quantified protein abundance changes, including those of GHs, in microbial communities in response to environmental and operational parameters [10–14]. Thus, it is feasible to consider that measuring the expression profiles of GHs or other relevant enzymes in ADs, and their changes over time, could provide information about the stable hydrolytic capabilities of a system and also diagnose potential causes of process failure such as variations in substrate availability [12, 15, 16]. Furthermore, the identification of specific GHs, or groups of them, as biomarkers of hydrolysis could be used to explore/indicate the carbohydrate solubilization capabilities of environmental microbial communities and to assess their potential for use as inocula in ADs or bioreactors.

Although informative, it is difficult to envision traditional global metaproteomic approaches as routine methods for monitoring abundance changes in a select group of enzymes; these endeavors are time-consuming and labor-intensive [17]. Thus, precise and sensitive alternatives that can provide faster decision-making capabilities regarding the hydrolytic potential of a microbial community either for use as inoculum or for adjustment of operational parameters in ADs are needed. As a point of reference, high-throughput technologies for the rapid screening of GH activities in different samples have been explored before. Some of these technologies have primarily used genetic information to screen for the presence of hundreds of GHs in complex environments [18], but these provide indirect evidence of metabolism. Some others have employed labeled protein approaches, antibody-based assays, or whole proteome interrogations to uncover the metabolic activities of microbial isolates or crude fungal broths [7, 15, 17], and hence are designed for specific deconstruction systems or require prior knowledge of the possible GHs present. To our knowledge, no information exists about the potential applicability of targeted proteomic approaches for the high-throughput profiling of enzyme groups such as GHs in microbiomes. In comparison to gene-based approaches that highlight the potential for lignocellulolytic metabolism in microbial communities, MS-based proteomic techniques provide information about the enzymes that are actively expressed by these communities. This is particularly important for biogas reactors, which contain a disproportionate number of microbial phyla that rely on the expression of few key enzymes or have minor groups within a community that are most active [12, 19–21].

**Fig. 1:**
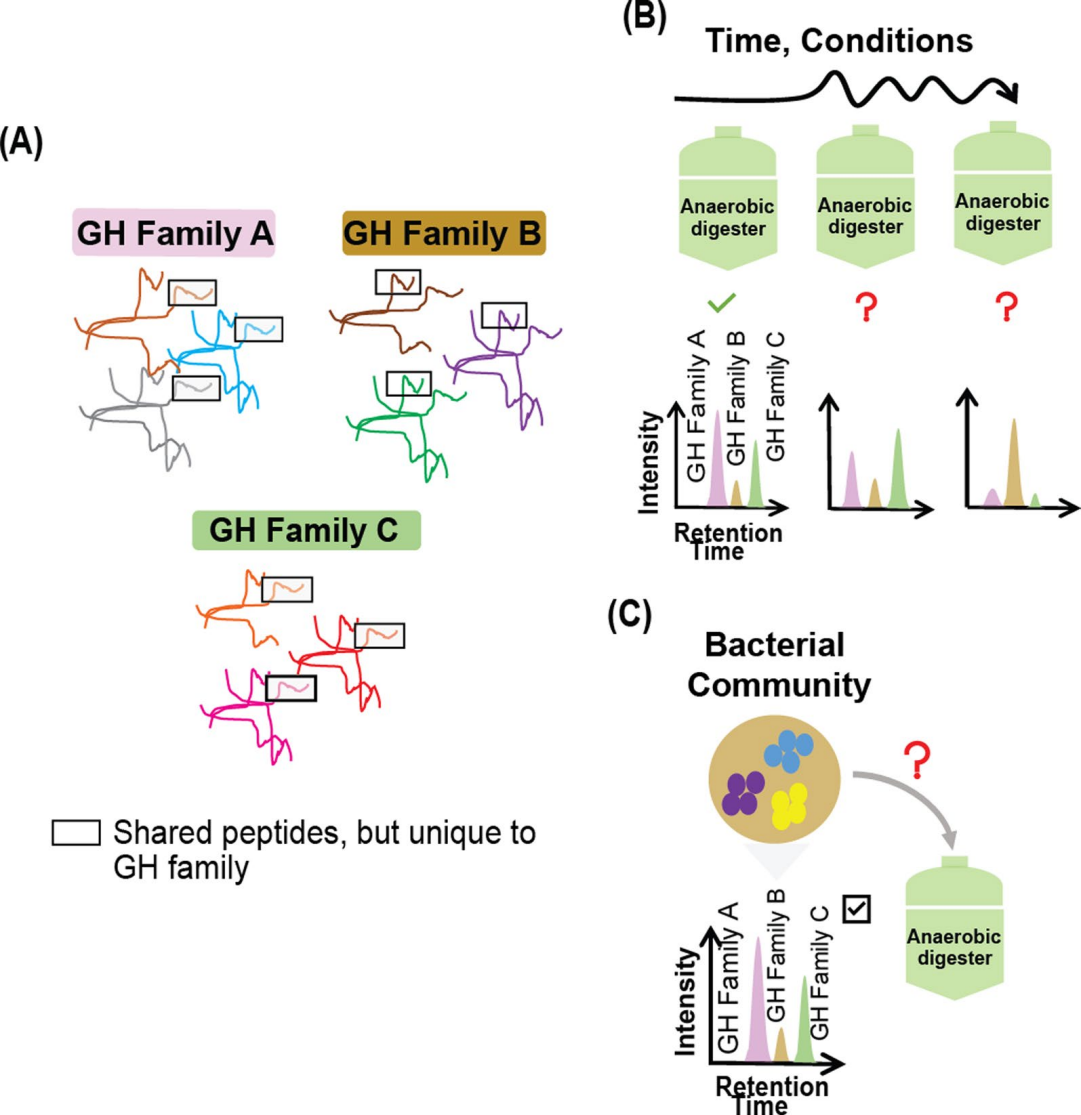
Selection of unique peptides for GH families in the development of a targeted proteomics assay. **A** In this paper, the in silico selection of shared peptides (in rectangles) within proteins from specific GH families that are otherwise unique to entire groups of them, was demonstrated. Potential applications are: **B** monitoring the stable hydrolytic capabilities of an anerobic digester or condition-dependent changes over time or **C** evaluation of the hydrolytic potential of a bacterial community extracted from the environment for use as the starting inoculum for a digester according to the expression of specific families of GHs

While global proteomic studies provide a broad, agnostic, quantitative interrogation of the entire range of measurable proteins in a sample, targeted proteomics focuses only on measurements of selected proteins of interest by using peptide sequences unique to those proteins in an organism [22, 23]. In contrast to discovery proteomics, targeted proteomic approaches are significantly faster and provide much greater sensitivity. These approaches allow for the direct measurement of a smaller, selective set of proteins of interest in a sample without the need for using substrate binding affinities, antibodies, or other activity-based probes that have been used before for certain carbohydrate-processing enzymes [24, 25]. However, despite the potential advantages of targeted proteomic approaches, their deployment in microbiomes is more complicated. Compared to single isolates, microbial communities are intrinsically complex, contain a much wider dynamic range of protein concentrations, and harbor extensive functional redundancy whereby multiple organisms perform the same function (i.e., by expressing functionally redundant proteins), making targeted protein measurements challenging. Therefore, targeted proteomic approaches for microbiomes, or “targeted metaproteomics”, require the adjustment of experimental design factors depending on the desired outcomes. Based on the research question, one of the adjustments is the selection of groups of peptides to identify/quantify a *specific category,* such as function or taxon, instead of a *single protein* [26]. Indeed, a recent study on ocean microbiomes demonstrated that it is possible to distinguish related marine cyanobacterial species by using a set of shared peptides from distinct protein biomarkers [27]. Thus, similar approaches can be developed for other complex microbiomes, such as those in ADs, with a focus on estimating the abundances of key enzymatic activities or microbes and monitoring the changes. To develop a targeted metaproteomics approach for diagnosing and monitoring the hydrolytic potential of microbial communities in ADs, the first step would be to identify unique peptides not for individual proteins but for protein populations performing related relevant functions such as specific GH families (Fig. 1) [28, 29]. Note that the initial objective here is the qualitative identification and tracking of GH families without a focus on absolute quantification, although tracking abundance changes might also be possible with this experimental approach.

Here, the feasibility and challenges of a targeted metaproteomic approach for monitoring key enzymes of carbohydrate deconstructing systems were evaluated. Taking an example of microbial communities within anaerobic digesters, we demonstrate an in silico peptide selection process for all proteins belonging to different GH families for a targeted analysis. To this end, we employed a dataset of 1401 high-quality (HQ) and medium–high quality (MHQ) metagenome-assembled genomes (MAGs) published by Campanaro et al., 2020 as part of the biogas microbiome project (https://biogasmicrobiome.env.dtu.dk/) [30]. This dataset consists of a comprehensive repository of microbial genomes representing the diversity found in different anaerobic digesters. By assembling an artificial bacterial community made of these microorganisms, the bioinformatics approach developed here identified discrete groups of shared peptides among proteins within a GH family which were unique compared to other background proteins, including other GHs and non-GH proteins. Although there were more than 500 shared peptides in each evaluated GH family, smaller numbers of these peptides could subset proteins in a GH family based on specific enzymatic activity and taxonomic origins. The presence of shared groups of peptides even in such a diverse and challenging microbiome, as tested here, is encouraging. These observations suggest the feasibility of this approach as a newer broad method for community activity screening and molecular-level performance monitoring in operational ADs, most of which will have substantially lower microbial diversity and complexity.

## Results and discussion

### Evaluation of the range of the taxonomic diversity and distribution of CAZymes in the known 1401 MAGs biogas microbiome dataset

To assess the feasibility of the targeted metaproteomic approach to identify and quantify specific functions in AD microbiomes, an extensive dataset was used. The 1401 HQ and MHQ metagenome-assembled genomes (MAGs) published by Campanaro et al. [30] provided a comprehensive framework of microorganisms commonly found in ADs. In total, 96% of MAGs were of bacterial origin, and the remaining 4% were of archaeal origin (Additional file 1: Fig. S2). Bacterial MAGs were grouped into 47 known phyla, while archaeal MAGs were clustered into six phyla (Additional file 1: Fig. S2). Importantly, not every member of a phylum in this dataset is expected to be found coexisting in a single digester. For example, the inoculum type and lignocellulosic material used as feedstock in ADs influence the occurrence and abundance of hydrolytic microbial species [5]. In other words, the taxonomic diversity derived from a more realistic system will be far less complex than the one used here. However, employing this comprehensive set of MAGs allowed us to explore the selection of unique tryptic peptides at a broader, more challenging level. The presence of unique peptides in this highly diverse community would support the applicability of this approach in lower complexity microbiomes.

**Fig. 2:**
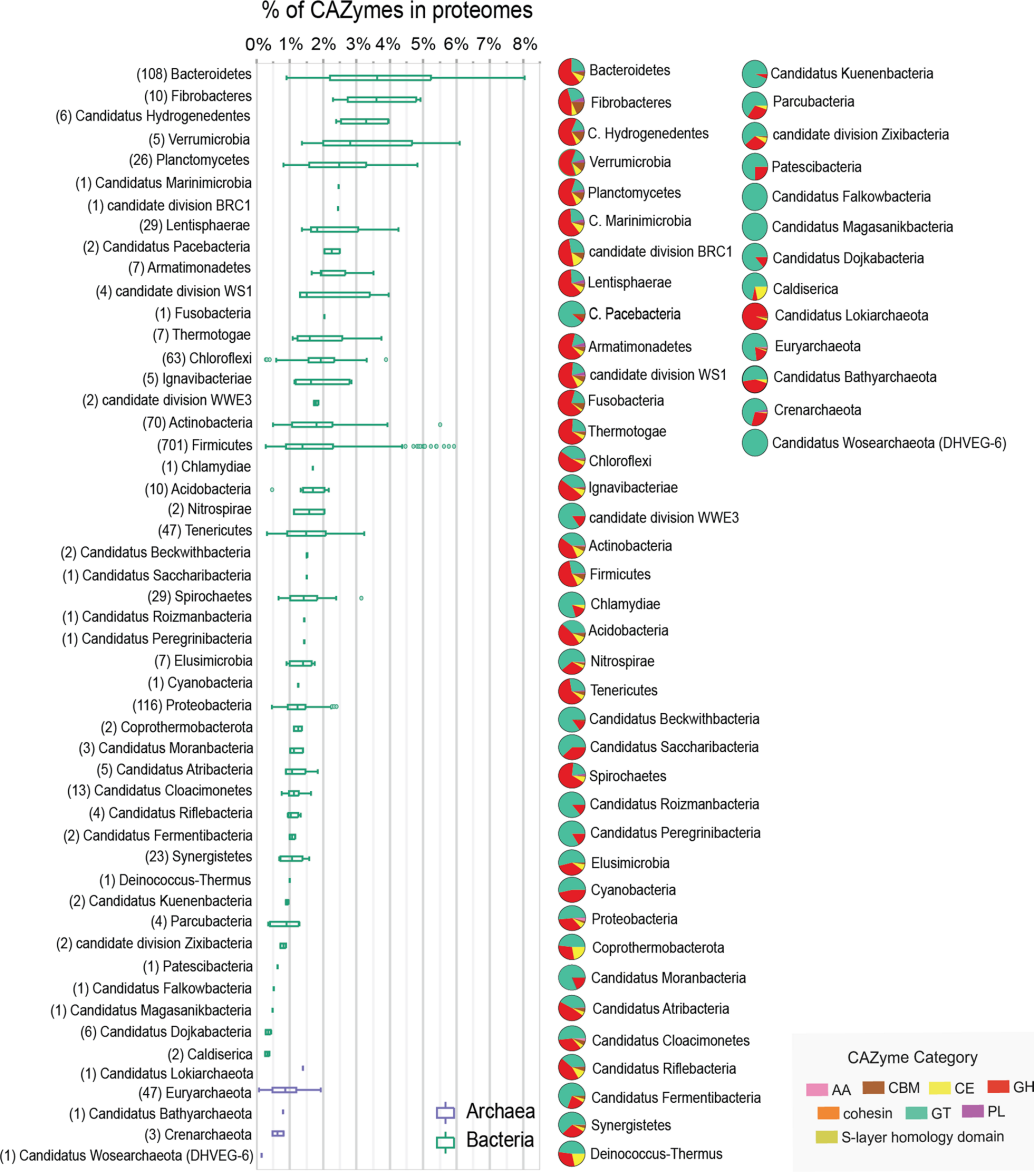
CAZymes annotated in the proteomes of different phyla. Box plots (left) show the percentage of CAZymes annotated in the proteomes of different bacterial and archaeal phyla using dbCAN2. The number of annotated MAGs per phylum are shown in parenthesis. Pie charts (right) show the relative fraction of different CAZyme classes, which include AAs (enzymes of the auxiliary activities), CBMs (carbohydrate-binding modules), CEs (carbohydrate esterases), GHs (glycoside hydrolases), GTs (glycosyltransferases), and PLs (polysaccharide lyases). Some proteins were also annotated with cohesin and S-layer homology domains, which are involved in the structure and formation of cellulosomes. MAGs lacking annotations at the phylum level are not shown

Among the bacterial phyla present in the dataset, several have been associated with the degradation of polysaccharides in ADs fed with lignocellulosic biomass, including members of the phyla *Firmicutes*, *Bacteroidetes*, *Fibrobacter*, *Spirochaetes*, and *Thermotogae* [5, 31] (Fig. 2). Others, including representatives from the lesser-known *Candidatus* Hydrogenedentes, *Armatimonadetes*, *Lentisphaerae*, and *Planctomycetes* phyla were present, which are potentially involved in the hydrolysis of polysaccharides [32]. Regarding archaeal MAGs, these were classified across six phyla, including the broad group of *Euryarchaeota*, which included the genera—*Methanobacterium*, *Methanosarcina*, *Methanoculleus*, and *Methanocorpusculum*, known to act in concert with hydrolytic bacteria to produce methane as the end product in ADs [33].

The number of proteins predicted in each MAG and the numbers of annotated CAZymes varied considerably (Fig. 2, Additional file 1: Fig. S3, Additional file 2: Table S1, Additional file 3: Table S2), reflecting the diverse metabolic specialization of different microbes during the anaerobic digestion of lignocellulose material. In total, 62,627 CAZymes were annotated across 1399 MAGs, while 2 MAGs lacked CAZyme annotation information according to our annotation criteria (see Materials and methods). The boxplots presented in Fig. 2 show the percentages of CAZymes present in the different protein databases or whole “proteomes” when grouped at the phylum level. The median percentage of CAZymes found per phylum was below 4%, which is consistent with the general abundance range of 1–3% of these enzymes from the total gene content of all living organisms and with the > 3% of the gene content of organisms with specialized functions such as the breakdown of complex carbohydrates found in lignocellulose [34–36]. Not surprisingly, MAGs from bacterial phyla *Bacteroidetes*, *Fibrobacteres*, *Verrumicrobia*, and *Planctomycetes*, which are known to degrade various complex carbohydrates from plant/algae material rich environments, were among the top phyla and had the highest median percentages of CAZymes in their proteomes (~ 2–4%). However, other less studied taxonomic groups, like the MAGs assigned to *Candidatus* Hydrogenedentes, *Candidatus* Marinimicrobia*,* and *candidate division BRC1,* also ranked high (~ 3%). The pie charts in Fig. 2 show that all these phyla have relatively higher proportions of carbohydrate degrading CAZymes—glycoside hydrolases (GHs), carbohydrate esterases (CEs), and polysaccharide lyases (PLs). In fact, more than 50% of the identified CAZymes in these phyla were GH proteins. This observation was also true for 11 other bacterial proteomes from phyla such as *Fusobacteria*, *Thermotogae,* and *Firmicutes*, which in biogas reactors are known to contribute to the utilization of complex carbohydrates, further highlighting the importance of these enzymes in the hydrolytic process [37].

CAZymes were also annotated in the proteomes of archaeal MAGs, with the median percentages by phylum being below 2% (Fig. 2). As opposed to bacterial phyla above, a large fraction of the annotated CAZymes in these archaeal phyla were glycosyl transferases (GTs), which are involved in the transfer of sugar moieties to specific acceptor molecules. Archaeal members are known to contain several genes expressing GTs in part due to their intricate protein N-glycosylation mechanisms, which are hypothesized to contribute to their ability to survive and adapt to harsh environments [38]. In fact, GT2 and GT4 families are known to predominate in Archaea [39] and was true for most members of the phyla *Euryarchaeota*, whose 47 MAGs captured here contained on average ten times as many GT2 or GT4 proteins compared to other GTs. The only exception was for a single archaeal MAG assigned to *Candidatus* Lokiarchaeota, whose 94% of identified CAZymes were GHs and the remaining were annotated to have carbohydrate-binding and CE domains. These observations agreed with prior metatranscriptomics analyses, which have shown the similar expression of GH ORFs in members of *Candidatus* Lokiarchaeota, and anaerobic utilization of carbohydrates has been described as one of their metabolic capacities [40].

The repertoire of CAZymes in biogas microbiomes identified above was used as the starting point in the targeted metaproteomic approach. As a proof of concept, the GH families with the highest number of representative proteins across the different MAGs in the biogas microbiome were selected as targets (Table 1), intentionally, to assess the selection of peptides from a very large number of proteins. However, depending on the system under study, other GH families can also be considered. Given the diverse biogas reactor sources that these MAGs were derived from, along with the diversity of the input lignocellulosic feedstock [30], the selected GH families (Table 1) covered a wide variety of important enzymatic functions. These ranged from the degradation of complex carbohydrates like endo-xylanases in hemicellulose (GH43) to those that cleave a variety of monosaccharides from polysaccharides and proteoglycans (GH2, GH13, GH3, and GH23) [5, 20]. The expression of these enzymes or enzymatic functions has also been reported before in other metaproteomic studies on biogas digesters, which further suggests their relevance in lignocellulolytic systems [12, 19, 41, 42]. These target GH families were then submitted to the bioinformatics pipeline described in Materials and methods in order to identify the minimum set of unique tryptic peptides that can describe/quantify them (Additional file 4: Table S3).

**Table 1:** GH families selected from the biogas microbiome data to test the in silico development of a minimum list of unique peptides able to differentiate them from other proteins

GH family	Enzymatic activities^a^	# of protein seeds across every MAG
13	Some enzymatic activities include: α-amylase, oligo-1,6-glucosidase, α-glucosidase, pullulanase, cyclomaltodextrinase, maltotetraose-forming α-amylase, isoamylase, dextran glucosidase, trehalose-6-phosphate hydrolase, among others acting on complex polysaccharides	4024
2	Most common activities include β-galactosidases, β-glucuronidases, β-mannosidases, exo-β-glucosaminidases and, in plants, a mannosylglycoprotein endo-β-mannosidase	2182
3	Exo-acting β-d-glucosidases, α-l-arabinofuranosidases, β-d-xylopyranosidases, *N*-acetyl-β-d-glucosaminidases, and *N*-acetyl-β-d-glucosaminide phosphorylases	2134
43	The major activities reported are α-l-arabinofuranosidases, endo-α-l-arabinanases (or endo-processive arabinanases), and β-d-xylosidases	1465
23	GHs in this family are lytic transglycosylases of both bacterial and bacteriophage origin and family G lysozymes of eukaryotic origin. Both of these enzymes are active on peptidoglycan, but only the lysozymes are active on chitin and chitooligosaccharides	1090

^a^Descriptions from CAZypedia.org (http://www.cazypedia.org/index.php?title=Main_Page&oldid=13510)

### A unique set of peptides can be selected to monitor GH families in the biogas microbiome

**Fig. 3:**
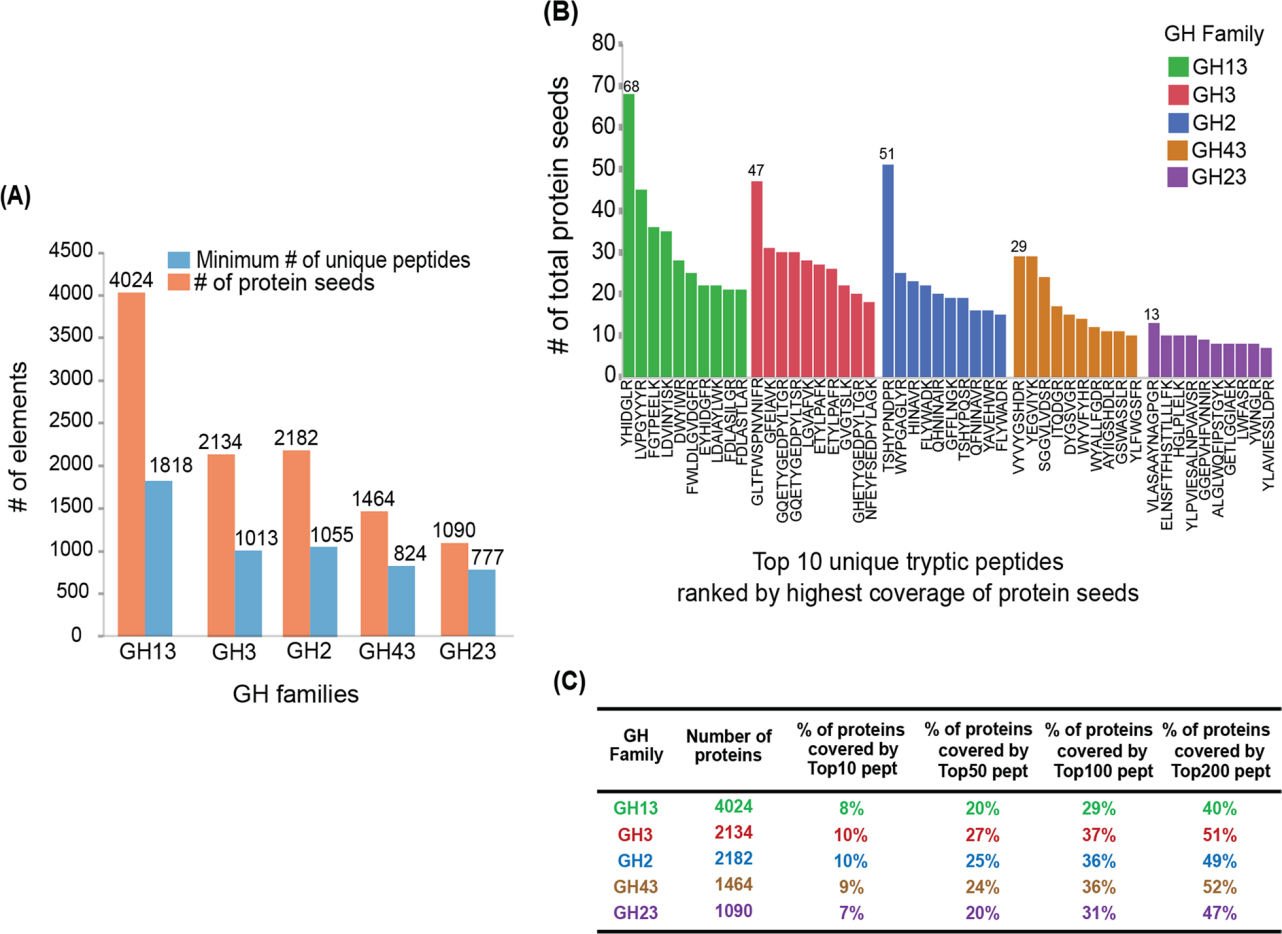
Minimum number of unique tryptic peptides and their associated number of protein seeds in targeted GH families. **A** Total number of unique peptides selected for each GH family after comparison against other proteins in the biogas microbiome dataset and the number of protein seeds in which they are found. **B** Top 10 tryptic peptides (from blue bars in **A**) ranked by the highest number of protein seeds they cover in each GH family. **C** Percentages of total proteins in GH families covered by top 10, 50, 100, and 200 peptides ranked by protein coverage

As observed in Fig. 3A, the total number of peptides required to measure all proteins for each of the five selected GH families, respectively, were around 800–2000. While this is a larger measurement set than would be normally possible on a triple-quadrupole mass spectrometer under targeted measurements (which is typically limited to 500–800 peptide targets), this range is within reach on the newer Orbitrap mass spectrometers that employ parallel reaction monitoring (PRM); in particular, new intelligent acquisition approaches on these MS platforms appear to allow this to be extendable even up to a few tens of thousands of peptides [43–46]. The maximum numbers of proteins covered by a peptide were 68, 47, 51, 29, and 13 in GH families 13, 3, 2, 43, and 23, respectively (Fig. 3B). Interestingly, we observed that different peptides selected within members belonging to the same families mapped to distinct domains in GHs and not always catalytic domains. Utilizing InterProScan searches [47], for example, in GH family 2, peptide TSHYPNDPR mapped to a GH2 catalytic domain (IPR006103) in the proteins it covered, whereas peptide WYPGAGLYR mapped to a GH2 sugar binding domain (IPR006104) in all the protein sequences it covered (Additional file 1: Fig. S4). As stated above, these peptides did not span catalytic motifs (Additional file 1: Fig. S4), which are usually conserved in both position and function [28]. In other words, the lack of conserved Lys and/or Arg residues, as well as the variability of other amino acids within these regions, may explain why more proteins were not covered by single tryptic peptides satisfying the empirical rules of peptide selection (i.e., 6–25 aa in length, etc.).

It should be noted that in this study, despite the community of organisms being quite extensive as it was assembled from all the published data, only ~ 200 peptides are theoretically sufficient to quantify the presence of ~ 50% proteins (equivalent to 545–2021 protein groups) from a GH family (Fig. 3C). We suspect that the microbial diversity of more defined biogas systems will be less complex, which would significantly reduce the number of tryptic peptides required to be monitored; thus, these results are encouraging and tractable for assaying and monitoring the capabilities of different microbial communities or keystone species within communities over time or across conditions in bioreactors.

The analytes identified for each of our targeted GH families also presented opportunities to investigate whether they could capture other relevant information within the respective family. In particular, we were interested in exploring the poly-specificity that members within the same GH families have to different substrates [28]. This is particularly relevant for ADs as, depending on the type of lignocellulosic material fed to them, sets of GHs with more specialized enzymatic activities may become more important for the successful degradation of complex polysaccharides and these enzymes frequently display affinities for more than one substrate [48].

### Unique tryptic peptides selected for groups of GHs can distinguish groups of proteins based on their enzymatic specificity

The majority of the GH families in the CAZy database are populated with enzymes having different substrate specificities. Such substrate specificity is usually expressed by the enzymatic commission numbers (EC) given to an enzyme [49]. The different substrate specificities of GHs have been suggested as an evolutionary divergence in these types of proteins, explained by the availability of carbohydrate metabolites with stereochemical resemblance to their original ones during evolution [29, 48]. For example, enzymes in the family GH3 are known to have dual or broad substrate specificities with respect to monosaccharide residues, linkage position, and chain length of the substrate [50]. This information led us to explore whether the identified peptides from selected families of GHs could also resolve groups of proteins within the same family with different enzymatic activities. To this end, we employed the GhostKOALA annotation pipeline [51] to retrieve EC numbers for all the GH proteins captured by the different peptides in the biogas microbiome (Additional file 4: Table S3).

**Fig. 4:**
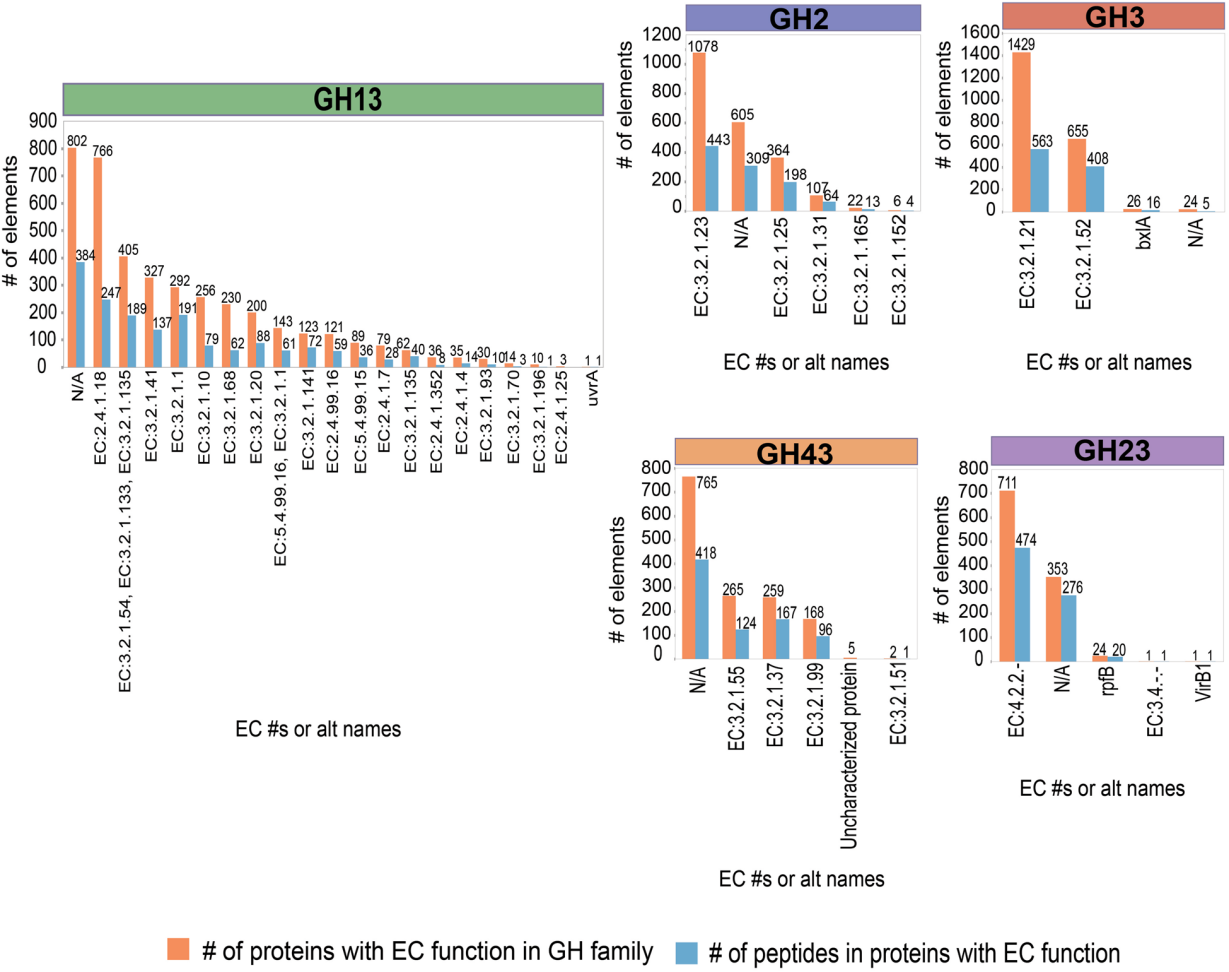
Functional classification of groups of proteins captured by the identified unique peptides for each GH family*.* Functional annotation of proteins captured by unique peptides was done with GhostKOALA. N/A = lacks annotation. A table of all EC numbers shown in the figure is presented in the Additional file 1: Table S4

Interestingly, we observed that the selected unique peptides grouped proteins within GH families based on different EC numbers (Fig. 4). For example, out of the 1055 peptides originally selected for family GH2, 443 captured GH2 proteins with beta-galactosidase activity (EC 3.2.1.23), while 196 were specific to GH2 proteins that have beta-mannosidase activity (EC.3.2.1.31). Even in the GH13 family, which contains ~ 30 different enzymatic specificities [52], discrete groupings of peptides and proteins according to EC numbers were observed. For example, to target GH13 proteins with amylo-(1,4 to 1,6)transglucosidase (EC 2.4.1.18) activity within the broad biogas microbiome studied here, only 247 peptides are necessary, while proteins annotated as cyclomaltodextrinases (EC 3.2.1.54), glucan 1,4-alpha-maltohydrolases (3.2.1.133) and neopullulanases (3.2.1.135) can be differentiated from other GH13 proteins by 189 peptides.

The differentiation given by the peptides selected here could be useful to target specific groups of GH proteins by substrate affinity in a bioreactor. This type of functional categorization of tryptic peptides within families of GHs has not been shown before and opens the possibility of monitoring enzymes dependent or independent of their families, but grouped under several EC numbers. These observations also encouraged us to continue exploring other types of categorizations given by the identified unique peptides, such as the taxonomic origins of the proteins. By determining which peptides are specific to certain phyla, a reduced number of peptides could in theory be used to target specific enzymatic activities from specific GH families produced from specific microbes.

### Unique tryptic peptides selected for groups of GHs can distinguish groups of proteins based on their taxonomic origins

In ADs, the hydrolytic ability of anaerobic bacteria for transforming polysaccharides into lower molecular weight intermediates that are used by other microbes during the anaerobic digestion food chain is a key element for their success [5]. Hence, we decided to use this data to categorize the peptides we selected and the proteins they mapped to based on their phylum-level origins. The phylum-level information of all MAGs annotated using dbCAN2 was taken from the biogas microbiome data from Campanaro et al. [30].

**Fig. 5:**
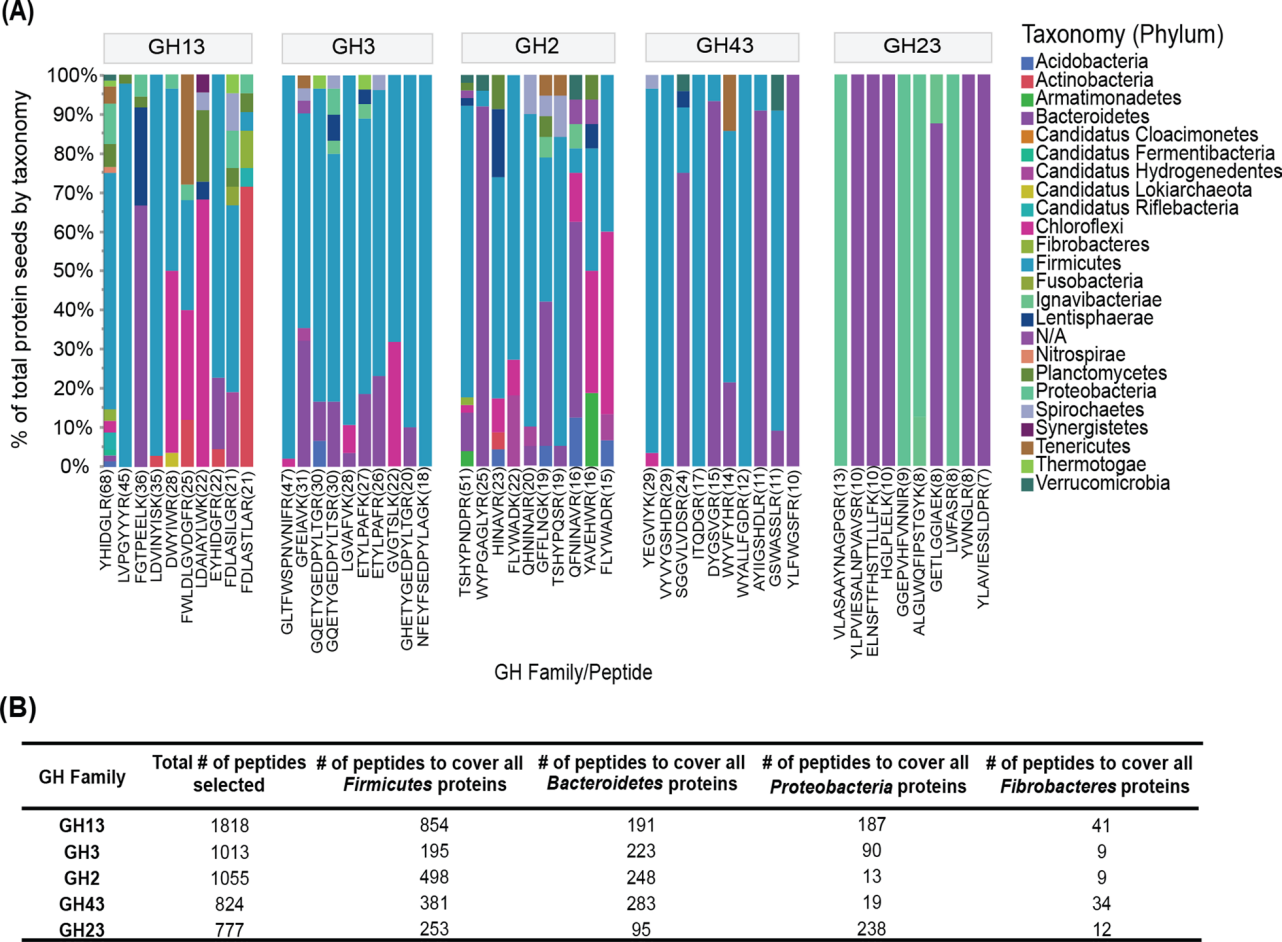
Classification of identified peptides by taxonomy using sequence information from the biogas microbiome MAGs. **A** Stacked bars show the classification of groups of proteins captured by the identified peptides, based on their taxonomical origins at the phylum level. Numbers in parenthesis are the total number of proteins in which the peptide is found. **B** Number of identified peptides required to cover all the proteins produced by members of a phylum for each GH family. NA, lacks annotation at the phylum level

Figure 5A shows the top 10 peptides (ranked by coverage of the number of proteins in Fig. 3B), and the phyla of the proteins they can quantify in each GH family. Although these peptides are still part of different domains of these proteins like those presented in Additional file 1: Fig. S4, it was interesting to observe the taxa distribution that they can capture. We noticed that some peptides captured proteins from different phyla, as seen in the broad functional groups of GH13, GH3, and GH2 families, while there were other peptides in families GH43 and GH23 that provided taxa-specific resolution. In family GH43, for example, peptides ITQDGR, VYVYGSHDR, WYALLFGDR were identified only in proteins from MAGs assigned to the *Firmicutes*, while peptide YLFWGSFR was specific to *Bacteroidetes* proteins. In family GH23, many more peptides covered proteins from single phyla; five of the top ten peptides mapped exclusively to proteins from *Bacteroidetes* and three of these GH23-specific peptides mapped only to proteins originated from *Proteobacteria*. However, these observations are mainly related to the total contribution of GH proteins per MAG/phylum, as the dataset used here has a greater representation of *Firmicutes*, *Proteobacteria*, and *Bacteroidetes* organisms (Additional file 1: Fig. S2).

From the total number of peptides selected for each GH family, we also calculated how many were necessary to cover all proteins related to phyla known for high hydrolytic potential in anaerobic environments (Fig. 5B) [5]. We observed that between 195 and 854 peptides are necessary to cover all *Firmicutes* proteins in each of the analyzed GH families, while the numbers were less for the other phyla analyzed. Importantly, because of how the selection of unique peptides was conceived in this study, these numbers include shared peptides across proteins with different taxonomic assignments. Thus, it is expected that these numbers could be less if one is only looking to target proteins from only a specific phylum. Indeed, alternative ways to develop the selection of unique peptides for GH families based on specific taxa can be planned. For example, if the goal is to monitor GH proteins from a specific taxonomic group (i.e., from a particular phylum) in a biological system, one could compare the phylum specific GH proteins against a background proteome comprising all remaining proteins from other phyla to identify relevant unique peptides instead of the broader taxa-agnostic comparisons described here.

The findings presented here are important from a microbiological point of view, as members of the hydrolytic *Firmicutes* and *Bacteroidetes* phyla are the most commonly found taxonomic groups in biogas plants [5]. The results suggest that it may be feasible to focus on GH proteins that are only derived from such keystone taxonomic groups and track their abundances within a complex community using a targeted metaproteomic approach.

## Conclusions

The focus of this work was to explore the in silico selection of a minimum set of peptides needed to exclusively target groups of proteins in GH families as a case study for the development of a targeted metaproteomic assay for identifying and quantifying these enzymes in anaerobic digesters (AD). Contrary to most traditional targeted proteomic workflows, in targeted metaproteomics, shared peptides across several proteomes specific to a category (such as a GH family) can be used to obtain relevant biological information about the system under study. Due to the reported hydrolytic functional redundancy found in bacterial communities thriving in biogas-producing reactors, we thought that it was more important to define unique peptides for a GH family instead of focusing on individual proteins or taxa.

During our analyses, we found that the number of tryptic peptides specific to GH families using sequence information derived from the biogas microbiome project range around 1000 in each case; nevertheless, ~ 200 peptides in each family were able to cover and hence identify ~ 50% of proteins belonging to each of the targeted GH families, which in most microbiomes would cover > 95% of a GH family abundance. These peptides can be further utilized to provide different degrees of functional distinction or taxonomical information, as demonstrated in this study. The number of tryptic peptides identified for the targeted GH proteins is expected to be lower in more representative or realistic datasets from biogas-producing reactors that contain substantially fewer members than the ones included here. Applied to defined microbiomes performing industrial processes, this approach can not only monitor the changes in the total abundance of key enzyme families like GHs, but also provide information about the contributing proteins, enzymatic activities, and microbes producing them; a level of resolution which is crucial for the control and monitoring of defined communities. Interestingly, this study was also useful to determine that it is possible to find unique peptides for individual GH proteins even within the same family (i.e., a GH3 protein versus another GH3) in a microbiome, like in more traditional targeted proteomic applications.

Targeted metaproteomics provides a way to identify and quantify proteins that can serve as indicators of the hydrolytic capacity or any other enzymatic activity of cellulolytic systems such as ADs. Currently, techniques that measure biodegradable organics present in the sludge fraction of a bioreactor (i.e., oxygen content, C/N ratios measurement) are employed to evaluate the performance of the anaerobic digestion process in faster ways, but these metrics lack molecular-level resolution and are comparatively less sensitive. In terms of protein abundance, the hydrolytic capacity of anaerobic digesters has been assessed by isolating active enzymes from different sample fractions and conducting in vitro substrate-degradation assays to characterize their enzymatic activity, but these do not reveal sequence-level identities of the CAZymes that are actively participating in the process and neither of their microbial origins [53, 54]. For families of GH proteins, targeted metaproteomic assays in ADs could be valuable to screen their expression or predict potential hydrolytic alterations based on changes to baseline or “stable operation” abundance values. In addition, these assays would be valuable for estimating molecular-level capabilities and responses of microbial communities to different substrates or conditions, which is a critical need in either building or utilizing constructed communities or defined cultures for bio-production. While we considered the worst-case scenario of a microbiome consisting of all known AD-related microbes, this assay would be most useful for more realistic lower complexity systems or assaying for specific activities within semi-defined microbiomes. The flexibility of these assays also allows them to be adapted to target other important CAZyme groups, such as carbohydrate esterases or methanogenesis enzymes. Additionally, such targeted characterizations could also be useful in health and nutrition, such as monitoring the levels of GHs in the rumen or human gut microbiome [34, 55], or be more widely applicable in any field interested in the study of carbohydrate metabolism. Importantly, in the human gut, alterations in the abundance levels of certain CAZymes have been linked to a number of diseases including Crohn’s disease, food allergies, colon cancer, amongst others [34, 56–58]. Furthermore, similar targeted metaproteomic approaches can also be adapted for monitoring abundances of biomarkers or key enzymatic activities during methanogenesis or anaerobic methane oxidation, which are crucial processes in the global geochemical cycles. Further investigations aiming to find peptides specific to groups of GHs for targeted metaproteomic applications could explore alternative avenues to reduce the potential number of candidates in them. These include, for example, the digestion of protein targets and background databases with enzymes other than trypsin that could exploit the sequence similarities of active site regions found in several GH families. Of note, after the initial in silico determination of a set of peptides, these analytes need to be tested experimentally to select the ones that can provide adequate signals in a mass spectrometer. This process will further reduce the list of initial peptide candidates, albeit at the expense of losing some proteins of interest.

## Materials and methods

### Re-processing of 1401 MAGs in dbCAN2 to assign CAZymes

Predicted genes and coding sequences (CDS) from 1401 bacterial and archaeal high-quality (HQ) [Completeness > 90%, Contamination < 5%] and medium–high quality (MHQ) [90% > Completeness ≥ 70%; 5% < Contamination < 10%] metagenome-assembled genomes (MAGs) reported in Campanaro et al. [30] were kindly provided by the first author of the study. The biogas microbiome project was a collaborative effort in which 134 published datasets (~ 0.9 Tbp sequence data) derived from a wide range of different biogas reactor systems (full-scale biogas plants and laboratory-scale bioreactors) fed with complex carbohydrates, proteins, and lipids, have been re-analyzed using comprehensive metagenome-centric analyses. The provided CDS were defined using Prodigal v2.6.2 ran in normal mode. The number of protein coding sequences from each MAG is provided in Additional file 2: Table S1. Identification of proteins with CAZymes’ domains was performed with the Carbohydrate-active enzyme Annotation (dbCAN2) meta server [59] for each MAG and used for downstream in silico analyses of unique peptides. CAZyme annotation included all major enzymatic categories in the CAZy database besides others like cohesin and S-layer homology domains which are structural components of bacterial cellulosomes [60]. The dbCAN2 searches were performed using the HMMER [61], DIAMOND [62], and Hotpep [63] tools. Proteins annotated by ≥ 2 tools were only considered to define CAZymes as per the software recommendations. HMMER annotations took priority over DIAMOND and Hotpep tools. In the cases where no HMMER annotation was obtained, common annotations between DIAMOND and Hotpep were only considered, otherwise, they were discarded. The number of proteins for each CAZyme class identified for each MAG is provided in Additional file 3: Table S2.

### Selection of “unique” tryptic peptides from GH families in the biogas microbiome MAGs

For selection of tryptic peptides unique to a GH family when compared to the rest of the microbiome, two sequence files were generated for each GH family of interest. For each of the highly represented GH families (targets), the corresponding protein sequences across all the MAGs were extracted from their original proteome files and subsequently clustered at 100% identity using CD-HIT v4.7 [64] to remove partial or fragmented sequences. For each target GH family, the protein sequences remaining in each MAG after removal of the GH family specific sequences were combined to create protein sequence backgrounds for selecting unique peptides. In contrast to traditional targeted proteomic approaches, it was essential to ensure that the selected tryptic peptides uniquely identify groups of proteins belonging to a GH family instead of individual proteins. As such, the uniqueness of each peptide candidate was compared to other non-GH bacterial and/or archaeal proteins, as well as to GH proteins belonging to other non-targeted GH families. Before generation of an initial list of tryptic peptides, the first 24 N-terminal amino acids from the clustered and targeted GH sequence seeds were removed using an in-house developed Python script to prevent utilization of potential signal peptides, which functionally get cleaved off in the protein maturation process. Target and background sequences were then digested in silico by trypsin using the prot2pept command of the Unipept [65] command line interface (CLI) (https://unipept.ugent.be/clidocs/prot2pept). The resulting tryptic peptides for the target GH family were then filtered following empirical rules of peptide selection [23, 66]. Several factors are usually considered to choose peptides for targeted proteomic experiments, which include MS properties, cleavage sites, and presence/absence of natural or chemically induced post-translational modifications. Since this demonstration was completely in silico, we emphasized peptide selection based on length (6–25 amino acids) and absence of residues with higher propensity towards artifactual modifications (i.e., Met or Cys). Hence, tryptic peptides having 6–25 amino acids without Met or Cys residues were only retained from the resultant target peptidomes so as to follow empirical targeted proteomic rules of peptide selection [23]. Peptidomes from targets and corresponding backgrounds were then compared using an in-house Python script, and unique peptides mapping only to the sequences (protein populations) of a target GH family were selected.

### Generation of a minimum list of unique tryptic peptides for GH families

A Python3 script was developed to select the minimum number of shared tryptic peptides between proteins in a GH family (peptides unique to the family). Briefly, the script takes the lists of unique tryptic peptides for distinct GH families obtained above and assembles groups of peptides and the corresponding proteins. The script then prioritizes groups with peptides mapping to the highest number of proteins and compares it against all other peptides–proteins groups in the list. To avoid consideration of already covered proteins into the counting of peptides to protein groups, the protein is then removed from the list of all the other peptides that map to it. These comparisons then repeat with the adjusted groups of proteins. In each successive iteration, peptides covering the largest number of proteins were selected until a final list of peptides covering all the proteins in the input list was obtained. Additional file 1: Fig. S1 provides an example depicting how the script works.

## Supplementary Information

**Additional file 1: Figure S1.** Example showing the process to select the minimum number of unique tryptic peptides and their associated number of protein seeds in different GH families. (A) Example of a starting list of seven proteins in a GH family each containing different numbers of peptides that are unique to a particular GH family and which can be shared or be unique among the proteins in that family. (B) The script first assembles groups of peptides and proteins and then orders them based on peptides capturing the greatest number of proteins. In this example, peptide 1 is shared among the majority of proteins in the input list. This group of 4 proteins that have peptide 1 is then compared to protein groups captured by other peptides. Based on this comparison, if a protein that has peptide 1 is found in a group with fewer number of proteins, the protein is removed from the list. In this case, proteins are removed from peptides 2 and 3, while peptide 4 loses all its proteins and is removed from further analysis. This iteration is repeated now with the second largest group of proteins sharing a peptide, which in this case is peptide 5. (C) Following this example, the final minimum list of “unique” peptides will have peptide 1 capturing four proteins (A, D, E, F), peptide 5 capturing two proteins (C, G) and either peptides 3 or 6 capturing one protein (B). **Figure S2.** Number of high quality (HQ) & medium high quality (MHQ) MAGs identified in the biogas microbiome project. MAGs were assigned to different phyla based on the tiered taxonomic assignment strategy described in the original paper by Campanaro et al., 2020. The inset shows the total percentages of MAGs per superkingdom. N/A- MAGs not assigned at the phylum level. **Figure S3**. Distribution of the sizes of the predicted proteomes from the HQ & MHQ quality MAGs from the biogas microbiome project*.* Coding sequences (CDS) were annotated using Prodigal v2.6.2. **Figure S4.** The identified tryptic peptides did not map to catalytic regions of the proteins. The figure shows examples of two GH2 protein sequences in the biogas microbiome dataset analyzed with InterProScan. According to our bioinformatic analysis, the unique tryptic peptides TSHYPNDPR and WYPGAGLYR are found in 51 and 25 different GH2 proteins, respectively. These numbers were among the highest numbers of proteins covered by single peptides in this family. As observed, these two peptides mapped to different GH2 family domains (highlighted in blue and purple colors). No tryptic peptides matching our selection criteria mapped to the active site motif in GH2 proteins (in yellow, PS00608) which is used as the signature pattern to classify GH proteins into this family. **Table S4.** Description of EC numbers shown in Fig. 4.

**Additional file 2: Table S1.** List of 1401 high quality and medium–high quality metagenome-assembled genomes (MAGs) from Campanaro et al. (2020) used for this study.

**Additional file 3: Table S2.** CAZy annotation results from the proteome of 1399 high quality and medium–high quality MAGs using the carbohydrate-active enzyme Annotation (dbCAN2) meta server. dbCAN2 searches were performed using HMMER, DIAMOND, and Hotpep tools.

**Additional file 4: Table S3.** Minimum number of tryptic peptides covering the highest number of proteins per GH family analyzed. Each peptide is specific to each GH family. GhostKOALA annotation results to get enzyme commission (EC) numbers for each protein are also shown.

## Data Availability

The MAGs files analyzed in this study were provided by Dr. Stefano Campanaro from Campanaro et al., 2020, as mentioned earlier. Custom scripts and all other data generated or analyzed during this study are included in this published article, its additional files, or available at the GitHub repository: https://github.com/pchirania/targeted_mp.

## Abbreviations

AD: Anaerobic digester
MAG: Metagenome-assembled genome
CAZyme: Carbohydrate active enzyme
GH: Glycoside hydrolase
CE: Carbohydrate esterase
PL: Polysaccharide lyase
HQ: High quality
MHQ: Medium–high quality
PRM: Parallel reaction monitoring
