# Supplementary material for: In silico evaluation of a targeted metaproteomics strategy for broad screening of cellulolytic enzyme capacities in anaerobic microbiome bioreactors
Source: Biotechnol Biofuels Bioprod. 2022 Mar 18;15:32. doi: 10.1186/s13068-022-02125-x (PMC8933973; doi:10.1186/s13068-022-02125-x)
Supplement: Supplementary file 1 — Additional file 1: Figure S1. Example showing the process to select the minimum number of unique tryptic peptides and their associated number of protein seeds in different GH families. (A) Example of a starting list of seven proteins in a GH family each containing different numbers of peptides that are unique to a particular GH family and which can be shared or be unique among the proteins in that family. (B) The script first assembles groups of peptides and proteins and then orders them based on peptides capturing the greatest number of proteins. In this example, peptide 1 is shared among the majority of proteins in the input list. This group of 4 proteins that have peptide 1 is then compared to protein groups captured by other peptides. Based on this comparison, if a protein that has peptide 1 is found in a group with fewer number of proteins, the protein is removed from the list. In this case, proteins are removed from peptides 2 and 3, while peptide 4 loses all its proteins and is removed from further analysis. This iteration is repeated now with the second largest group of proteins sharing a peptide, which in this case is peptide 5. (C) Following this example, the final minimum list of “unique” peptides will have peptide 1 capturing four proteins (A, D, E, F), peptide 5 capturing two proteins (C, G) and either peptides 3 or 6 capturing one protein (B). Figure S2. Number of high quality (HQ) & medium high quality (MHQ) MAGs identified in the biogas microbiome project. MAGs were assigned to different phyla based on the tiered taxonomic assignment strategy described in the original paper by Campanaro et al., 2020. The inset shows the total percentages of MAGs per superkingdom. N/A- MAGs not assigned at the phylum level. Figure S3. Distribution of the sizes of the predicted proteomes from the HQ & MHQ quality MAGs from the biogas microbiome project. Coding sequences (CDS) were annotated using Prodigal v2.6.2. Figure S4. The identified tryptic peptides did not map t [file 13068_2022_2125_MOESM1_ESM.docx]

**Additional File 1**

***In-silico* evaluation of a targeted metaproteomics strategy for broad screening of cellulolytic enzyme capacities in anaerobic microbiome bioreactors**

Manuel I. Villalobos Solis^1#^, Payal Chirania^1,2#^, Robert L. Hettich^1^*

^1^ Biosciences Division, Oak Ridge National Laboratory, Oak Ridge, Tennessee 37831, USA

^2^ UT-ORNL Graduate School of Genome Science and Technology, University of Tennessee, Knoxville, Tennessee 37996, USA

^#^These authors contributed equally to this work

*Corresponding author

**Correspondence:**

Robert L. Hettich

Oak Ridge National Lab, Oak Ridge, TN 37831

Email: [hettichrl@ornl.gov](mailto:hettichrl@ornl.gov)

Phone: 865-574-4968


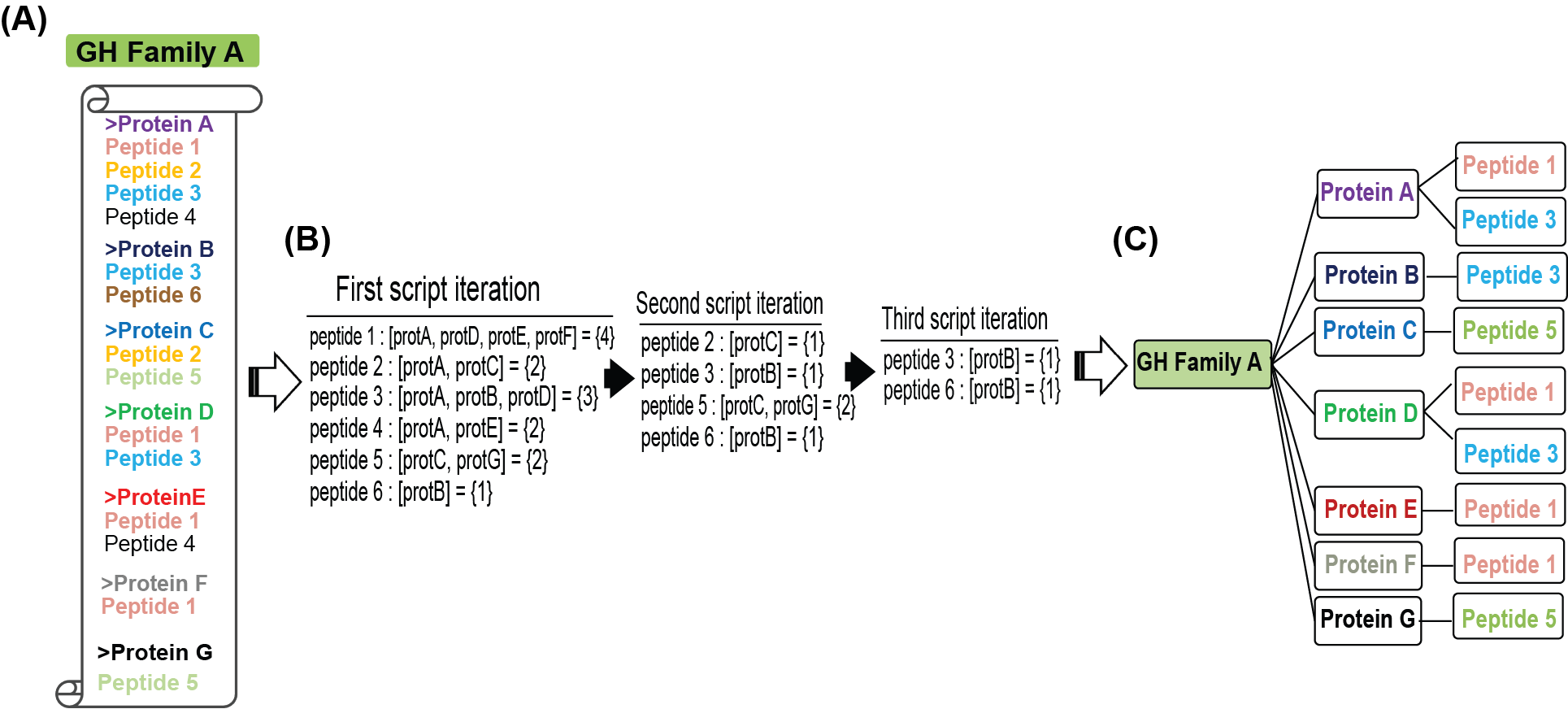


**Figure S1. Example showing the process to select the minimum number of unique tryptic peptides and their associated number of protein seeds in different GH families. (A)** Example of a starting list of seven proteins in a GH family each containing different numbers of peptides that are unique to a particular GH family and which can be shared or be unique among the proteins in that family. **(B)** The script first assembles groups of peptides and proteins and then orders them based on peptides capturing the greatest number of proteins. In this example, peptide 1 is shared among the majority of proteins in the input list. This group of 4 proteins that have peptide 1 is then compared to protein groups captured by other peptides. Based on this comparison, if a protein that has peptide 1 is found in a group with fewer number of proteins, the protein is removed from the list. In this case, proteins are removed from peptides 2 and 3, while peptide 4 loses all its proteins and is removed from further analysis. This iteration is repeated now with the second largest group of proteins sharing a peptide, which in this case is peptide 5. **(C)** Following this example, the final minimum list of “unique” peptides will have peptide 1 capturing four proteins (A, D, E, F), peptide 5 capturing two proteins (C, G) and either peptides 3 or 6 capturing one protein (B).

**
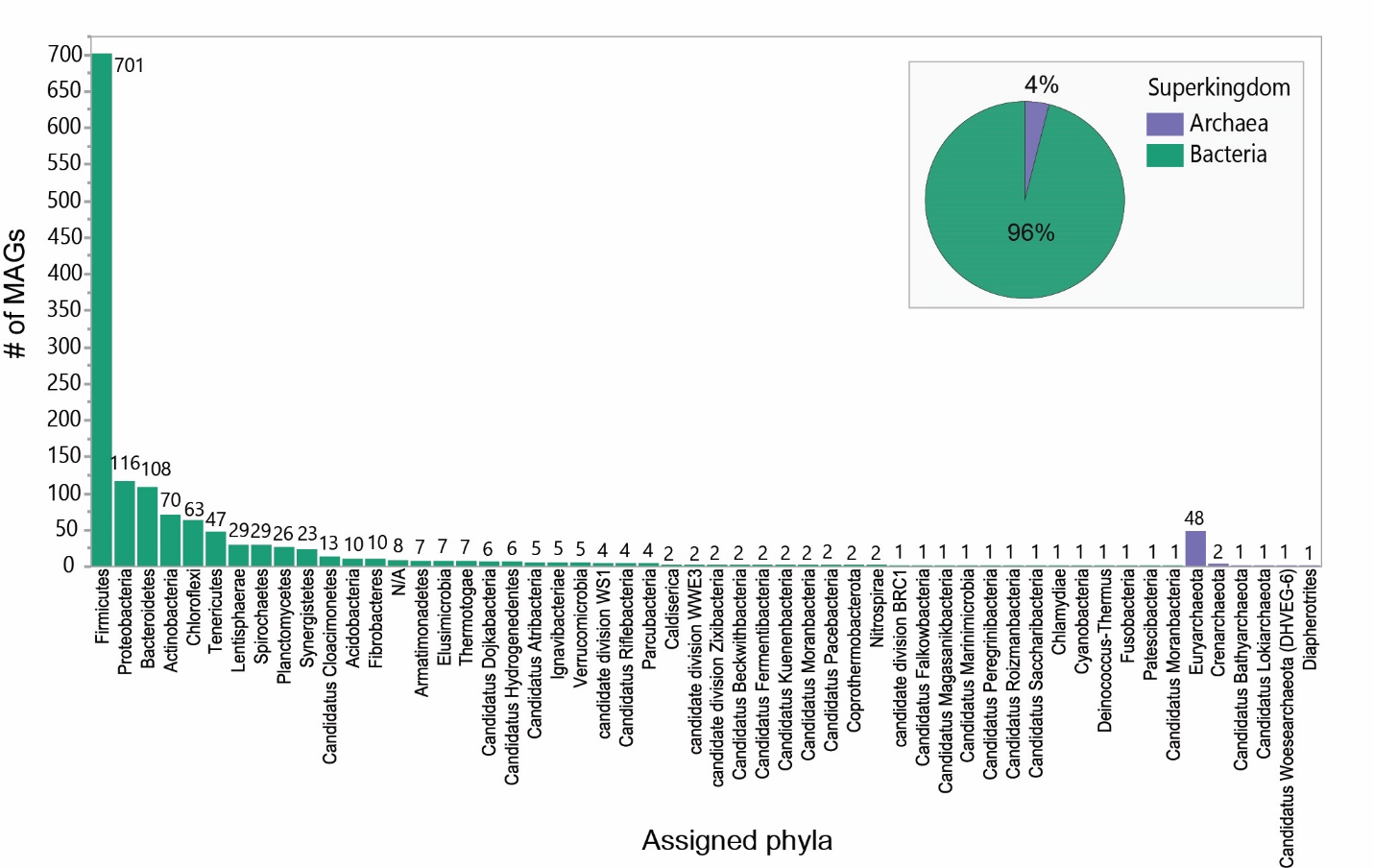
**

**Figure S2. Number of high quality (HQ) & medium high quality (MHQ) MAGs identified in the biogas microbiome project.** MAGs were assigned to different phyla based on the tiered taxonomical assignment strategy described in the original paper by Campanaro *et al*., 2019. The inset shows the total percentages of MAGs per superkingdom. N/A- MAGs not assigned at the phylum level.

**
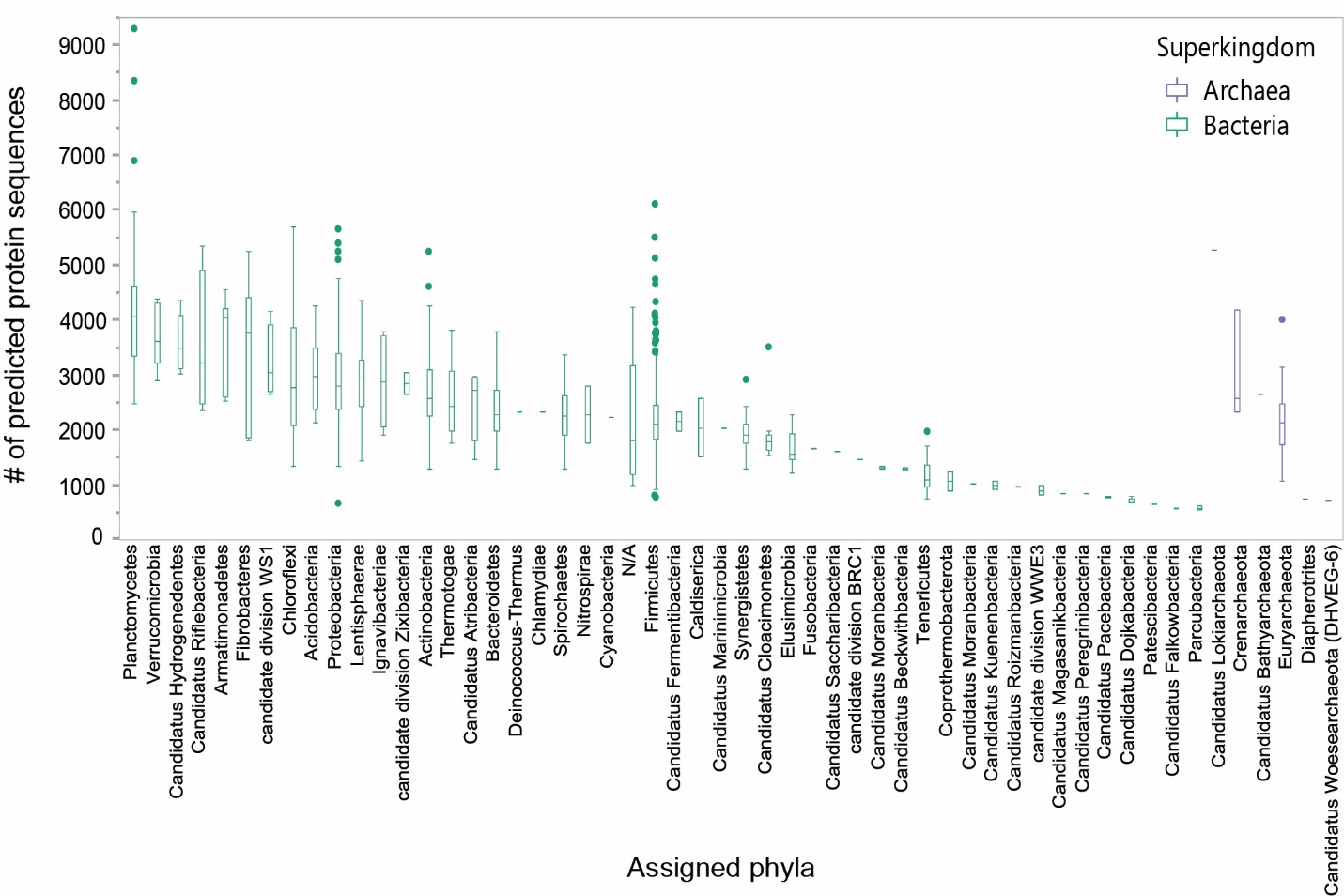
**

**Figure S3. Distribution of the sizes of the predicted proteomes from the HQ & MHQ quality MAGs from the biogas microbiome project***.* Coding sequences (CDS) were annotated using Prodigal v2.6.2.


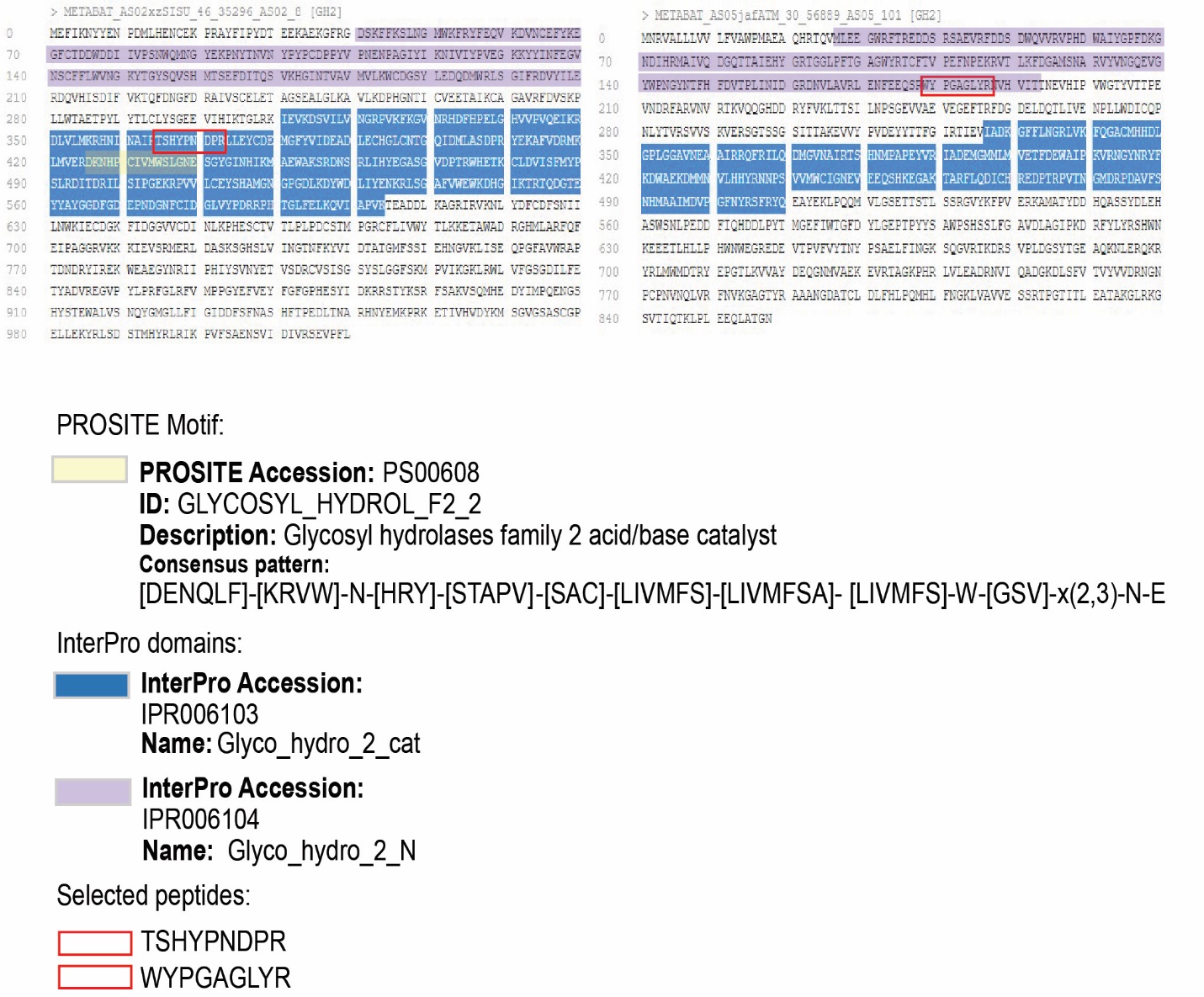


**Figure S4. The identified tryptic peptides did not map to catalytic regions of the proteins.** The figure shows examples of two GH2 protein sequences in the biogas microbiome dataset analyzed with InterProScan. According to our bioinformatic analysis, the unique tryptic peptides TSHYPNDPR and WYPGAGLYR is found in 51 and 25 different GH2 proteins, respectively. These numbers were among the highest numbers of proteins covered by single peptides in this family. As observed, these two peptides mapped to different GH2 family domains (highlighted in blue and purple colors). No tryptic peptides matching our selection criteria mapped to the active site motif in GH2 proteins (in yellow, PS00608) which is used as the signature pattern to classify GH proteins into this family.

**Table S4.** Description of EC numbers shown in Figure 4.

| **EC Number /alternative names** | **Description** |
| --- | --- |
| EC 2.4.1.18 | 1,4-alpha-glucan branching enzyme |
| EC 3.2.1.54 | **Cyclomaltodextrinase** |
| EC 3.2.1.133 | **Glucan 1,4-alpha-maltohydrolase** |
| EC 3.2.1.135 | **Neopullulanase** |
| EC 3.2.1.41 | **Pullulanase** |
| EC 3.2.1.1 | **Alpha-amylase** |
| EC 3.2.1.10 | **Oligo-1,6-glucosidase** |
| EC 3.2.1.68 | **Isoamylase** |
| EC 3.2.1.20 | **Alpha-glucosidase** |
| EC 5.4.99.16 | **Maltose alpha-D-glucosyltransferase** |
| EC 3.2.1.141 | **4-alpha-D-((1->4)-alpha-D-glucano)trehalose trehalohydrolase** |
| EC 2.4.99.16 | **Starch synthase (maltosyl-transferring)** |
| EC 2.4.99.15 | **(Kdo)(3)-lipid IV(A) (2-4) 3-deoxy-D-manno-octulosonic acid transferase** |
| EC 2.4.1.7 | **Sucrose phosphorylase** |
| EC 3.2.1.135 | **Neopullulanase** |
| EC 2.4.1.352 | **Glucosylglycerate phosphorylase** |
| EC 2.4.1.4 | **Amylosucrase** |
| EC 3.2.1.93 | **Alpha,alpha-phosphotrehalase** |
| EC 3.2.1.70 | **Glucan 1,6-alpha-glucosidase** |
| EC 3.2.1.196 | **Limit dextrin alpha-1,6-maltotetraose-hydrolase** |
| EC 2.4.1.125 | **Sucrose--1,6-alpha-glucan 3(6)-alpha-glucosyltransferase** |
| uvrA | **excinuclease ABC subunit A** |
| EC 3.2.1.23 | **Beta-galactosidase** |
| EC 3.2.1.25 | **Beta-mannosidase** |
| EC 3.2.1.21 | **Beta-glucosidase** |
| EC 3.2.1.31 | **Beta-glucuronidase** |
| EC 3.2.1.165 | **Exo-1,4-beta-D-glucosaminidase** |
| EC 3.2.1.152 | **Mannosylglycoprotein endo-beta-mannosidase** |
| EC 3.2.1.52 | **Beta-N-acetylhexosaminidase** |
| bxlA | **Xylan 1,3-beta-xylosidase** |
| EC 3.2.1.55 | **Non-reducing end alpha-L-arabinofuranosidase** |
| EC 3.2.1.37 | **Xylan 1,4-beta-xylosidase** |
| EC 3.2.1.99 | **Arabinan endo-1,5-alpha-L-arabinosidase** |
| EC 3.2.1.51 | **Alpha-L-fucosidase** |
| EC 4.2.2.- | **Lyases** |
| rpfB | **resuscitation-promoting factor RpfB** |
| EC 3.4.-.- | **Peptidases** |
| virB1 | **type IV secretion system protein VirB1** |
